# Data in support of high rate of pregnancy related deaths in Maiduguri, Borno State, Northeast Nigeria

**DOI:** 10.1016/j.dib.2018.03.038

**Published:** 2018-03-16

**Authors:** Patience I. Adamu, Muminu O. Adamu, Hilary I. Okagbue

**Affiliations:** a Department of Mathematics, Covenant University, Canaanland, Ota, Nigeria; b Department of Mathematics, University of Lagos, Akoka, Lagos, Nigeria

**Keywords:** Pregnancy related deaths, Dataset, Maternal death, Maternal mortality, Statistics, Pregnancy, Maiduguri

## Abstract

Pregnancy related deaths (PRD) are public health concern in most developing countries and Nigeria in particular. Despite the efforts put in by the concerned authorities, PRD remains an integral part of maternal mortality or maternal deaths in Nigeria in general and Borno state in particular, as evidenced from the records obtained from Umaru Shehu Hospital, Maiduguri (a state hospital in the state capital. The data contains frequency of PRD in months and grouped into gynaecology, ante-natal and post-natal, and labour obtained from mid-2009 to mid - 2017. The statistical analysis of the data may reveal the extent of incidence or epidemiology of PRD is in the state.

**Specification Table**

**Table:** 

**Subject area**	Medicine
**More specific subject area**	Maternal mortality, survival analysis, epidemiology, biostatistics
**Type of data**	Tables and figures
**How data was acquired**	Unprocessed secondary data
**Data format**	Filtered and partially analysed
**Experimental factors**	Nil
**Experimental features**	Descriptive statistics of the occurrence of pregnancy related deaths
**Data source location**	Umaru Shehu Hospital, Maiduguri, Borno State, Nigeria
**Data accessibility**	All the data are in this data article
**Software**	SPSS Statistical program and Microsoft Excel

**Value of the data**

• The data can be helpful in the estimation of maternal mortality rate in the state.
• The data can be useful for policy makers to monitor health policies and implementation as it concerns maternal and child health.
• The data can be useful in monitoring the health indices of the Millennium Development Goals and Sustainable Development Goals of the United Nations.
• The data is useful in medical education and epidemiological studies.
• The data is an indicator of the adverse effects of current insurgency in the state with high pregnancy related deaths.
• Several known statistical models can be applied to the data such as missing data analysis, time series, correlation, regression analysis, logistic regression.
• The data is reusable, can be extended to include a longitudinal study or health assessment studies [1], [2], [3], [4], [5], [6], [7], [8].

## 1 Data

The data contains pregnancy related deaths (PRD) records grouped into gynaecology, ante-natal, post-natal, and labour related deaths of pregnant women obtained from June 2009 to July 2017 and collected from the hospital records of Umaru Shehu hospital, Maiduguri, Borno state, Nigeria. The data is quantitative in nature and presented the frequency of PRD in months. The data are shown in Table 1, Table 2, Table 3, Table 4, Table 5, Table 6, Table 7, Table 8, Table 9 as monthly frequencies of the recorded PRD.

**Table 1:** PRD recorded at the hospital from June 2009 to December 2009.

Month/PRD	Jun	Jul	Aug	Sep	Oct	Nov	Dec	Total
Gynaecology	1	5	2	3	2	4	–	17
Ante/post-natal	16	1	14	4	3	8	2	48
Labour	1	2	3	2	1	1	1	11
Total	18	8	19	9	6	13	3	76

**Table 2:** PRD recorded at the hospital from January 2010 to December 2010.

Month/PRD	Jan	Feb	Mar	Apr	May	Jun	Jul	Aug	Sep	Oct	Nov	Dec	Total
Gynaecology	2	–	–	–	–	–	3	–	–	1	–	3	9
Ante/post-natal	–	3	3	2	4	4	5	1	–	1	–	–	23
Labour	3	2	2	1	1	1	1	–	–	–	–	1	12
Total	5	5	5	3	5	5	9	1	–	2	–	4	44

**Table 3:** PRD recorded at the hospital from January 2011 to December 2011.

Month/PRD	Jan	Feb	Mar	Apr	May	Jun	Jul	Aug	Sep	Oct	Nov	Dec	Total
Gynaecology	–	–	2	–	2	–	–	–	–	–	1	–	5
Ante/post-natal	1	1	6	4	10	–	1	1	–	–	–	2	26
Labour	–	1	–	1	2	4	–	–	1	–	3	–	12
Total	1	2	8	5	14	4	1	1	1	–	4	2	43

**Table 4:** PRD recorded at the hospital from January 2012 to December 2012.

Month/PRD	Jan	Feb	Mar	Apr	May	Jun	Jul	Aug	Sep	Oct	Nov	Dec	Total
Gynaecology	3	1	–	1	–	–	1	–	–	1	–	1	8
Ante/post-natal	1	–	2	1	–	–	–	1	–	–	–	3	8
Labour	1	2	–	–	1	1	1	4	2	5	1	–	18
Total	5	3	2	2	1	1	2	5	2	6	1	4	34

**Table 5:** PRD recorded at the hospital from January 2013 to December 2013.

Month/PRD	Jan	Feb	Mar	Apr	May	Jun	Jul	Aug	Sep	Oct	Nov	Dec	Total
Gynaecology	–	–	–	2	–	2	–	1	–	–	–	–	5
Ante/post-natal	–	–	–	–	2	4	1	–	4	4	–	–	15
Labour	1	5	1	–	1	–	–	1	–	–	1	–	10
Total	1	5	1	2	3	6	1	2	4	4	1	–	30

**Table 6:** PRD recorded at the hospital from January 2014 to December 2014.

Month/PRD	Jan	Feb	Mar	Apr	May	Jun	Jul	Aug	Sep	Oct	Nov	Dec	Total
Gynaecology	–	–	5	–	1	2	–	–	3	4	–	–	15
Ante/post-natal	1	–	1	2	5	7	2	–	5	5	7	1	36
Labour	–	–	–	–	1	–	–	1	1	–	1	–	4
Total	1	–	6	2	7	9	2	1	9	9	8	1	55

**Table 7:** PRD recorded at the hospital from January 2015 to December 2015.

Month/PRD	Jan	Feb	Mar	Apr	May	Jun	Jul	Aug	Sep	Oct	Nov	Dec	Total
Gynaecology	–	–	–	2	–	3	–	1	2	6	2	–	16
Ante/post-natal	1	2	2	5	7	4	1	1	7	4	6	1	41
Labour	2	–	–	1	2	–	–	1	1	–	1	2	10
Total	3	2	2	8	9	7	1	3	10	10	9	3	67

**Table 8:** PRD recorded at the hospital from January 2016 to December 2016.

Month/PRD	Jan	Feb	Mar	Apr	May	Jun	Jul	Aug	Sep	Oct	Nov	Dec	Total
Gynaecology	2	–	5	5	–	–	–	3	3	1	–	–	19
Ante/post-natal	1	–	–	7	3	6	5	–	3	6	5	1	37
Labour	–	–	–	–	–	–	1	1	–	–	–	–	2
Total	3	–	5	12	3	6	6	4	6	7	5	1	58

**Table 9:** PRD recorded at the hospital from January 2017 to July 2017.

Month/PRD	Jan	Feb	Mar	Apr	May	Jun	Jul	Total
Gynaecology	1	1	1	2	–	–	–	5
Ante/post-natal	1	–	–	3	4	2	3	13
Labour	–	–	–	–	–	2	1	3
Total	2	1	1	5	4	4	4	21

## 2 Experimental design, methods and materials

### 2.1 Data collection

The data was collected from the hospital records of the Obstetrics and Gynaecology unit of the Umaru Shehu hospital, Maiduguri, Borno state, Nigeria. The data was collected manually by books of records. The data is presented as monthly frequencies of the recorded PRD.

### 2.2 Description of the hospital

The hospital is a key state owned, secondary health care facility that receives referred cases from primary healthcare centers and transfers specialized cases to the University of Maiduguri Teaching Hospital. The hospital is very vital in health care delivery and consultancy services in the state for the following reasons:

a) It is one of few public owned hospitals in the state.
b) It is connected to the various primary health care facilities in the state.
c) It is located in the state capital which is firmly in the control of the Nigerian military fighting against Boko haram insurgency in the state.
d) It serves as key health care service centre for those displaced by the Boko haram insurgents known as internally displaced persons (IDPs).
e) It serves as one of the hospitals were victims of Boko haram insurgency are treated such as victims of bomb blasts, rape, forced marriage, wounded soldiers and civilians caught in the war and so on.
f) It serves the state capital and environs, especially now that most people are living there because of the ongoing war against Boko haram insurgency.

### 2.3 Detailed data description

Nigeria is divided into six geo-political zones. The north east zone contains the states of Adamawa, Bauchi, Borno, Gombe, Taraba and Yobe.

The data was obtained from one of the aforementioned and renowned state hospitals in Borno state which is a state from the northeast region of the country that historically has high maternal mortality and now worsened by the current Boko haram insurgency.

Borno state is located in the Northeast Nigeria, occupies an area of approximately 22, 316 square miles and mostly inhabited by the Kanuri people. The increase in pregnancy related deaths (PRD) as shown in the data is an indication of the adverse effect of the Boko haram insurgency on the health of the indigenes of the state because as stated earlier, the hospital serves a greater percentage of the populace especially those displaced by the ongoing insurgency. The total population that the hospital serves cannot be accurately quantified because IDPs are flooded in the state capital but the population of the state capital is 758,700 [10].

Moreover, the hospital data on the total patients that visited for consultancy services or treatment are missing due to poor records keeping. The hospital officers attributed that to the structure of the hospital which operates a decentralized model of records keeping. The records are kept and maintained at the different units of the hospital.

### 2.4 Pregnancy related deaths classification

The cause of the PRD of the raw data as seen was grouped into three; namely; Gynaecology, ante-natal and post-natal and death during labour. The groupings are deliberate attempts to classify PRD into three major kinds. The reliability of the data is to discretize PRD into exclusive and exhaustive classes. This comes as an attempt to estimate actual maternal mortality in a low income country like Nigeria where records of births and deaths are often incomplete or nonexistent. However, generally, there seems to be different classifications of PRD or the fatality of pregnancy related disorders. The list of the classifications and the specific diseases are not exhaustive. The classifications are given regardless of the case fatality rate of each disorder: These are shown in Table 10, Table 11, Table 12, Table 13.

**Table 10:** Maternal related problem (gynaecological).

	Disease/disorders	Example
1	Hypertensive disorders	Chronic hypertension, Preeclampsia, Eclampsia. Gestational hypertension, Eclampsia superimposed.
2	Gestational diabetes.	
3	Anaemia	Sickle cell related.
4	Deep vein thrombosis.	
5	Heart diseases	Peripartum Cardiomyopathy, Eisenmenger's syndrome.
6	Hypercoagulability	
7	Infection	Influenza, Hepatitis E, Herpes simplex, measles, Human African Trypanosomiasis, Varicella.
8	Endocrine disorders	Thyroid diseases, hypothyroidism, neurological Cretinism, Diabetes Mellitus.
9	Automotive diseases	Celiac disease, Systemic Lupus erythematous, Behçet's disease, multiple Sclerosis, Sepsis.
10	Others	Cancer, Cirrhosis, kidney disorders, mental and respiratory disorders.

**Table 11:** Fetal and placental disorders (ante-natal).

	Disease/disorders	Example
1	Multiple and Ectopic pregnancy	
2	Placental abruption.	
3	Vertically transmitted infection	Cytomegalovirus, Neonatal Herpes simplex, Rubella, Toxoplasmosis, Human papillomavirus, Urea plasma urealyticum, chicken pox, Coxasacklevirus, Chlamydia, HIV, Staphylococcus aureus, Gonorrhea, Syphilis, Hepatitis B virus, Human T-lymphtrophic virus.
4	Others

**Table 12:** Post-natal disorders.

	Disease/disorders	Example
1	Hemorrhage	Postpartum bleeding
2	Postnatal trauma	Depression, anxiety
3	Others

**Table 13:** Labour related deaths.

	Disease/disorders
1	Unskilled personnel and inadequate tools required for child delivery.
2	Complications during Caesarean section.
3	Delivery with no one present
4	Limited choice of delivery.
5	Underage child pregnancy
6	Others
